# Network analysis of influential risk factors in adolescent suicide attempters

**DOI:** 10.1186/s13034-024-00842-9

**Published:** 2024-11-25

**Authors:** Jennifer Fernandez-Fernandez, Luis Jiménez-Treviño, Jorge Andreo-Jover, Wala Ayad-Ahmed, Teresa Bobes Bascarán, Manuel Canal-Rivero, Annabel Cebria, Benedicto Crespo-Facorro, Alejandro De la Torre-Luque, Marina Diaz-Marsa, Ana Gonzalez-Pinto, Sandra Gomez, Iría Grande, Noelia Iglesias, Francisco Ortin, Katya March, Angela Palao, Iván Pérez-Díez, Carla Pérez-Guerra, Miguel Ruiz-Veguilla, Eduard Vieta, Victor Perez-Sola, Pilar Alejandra Saiz

**Affiliations:** 1 Department of Psychiatry, University of Oviedo, Oviedo, Spain; 2 Instituto de Investigación Sanitaria del Principado de Asturias (ISPA), Oviedo, Spain; 3 Instituto Universitario de Neurociencias del Principado de Asturias (INEUROPA), Oviedo, Spain; 4 Centro de Investigación Biomédica en Red de Salud Mental (CIBERSAM), Madrid, Spain; 5 Servicio de Salud del Principado de Asturias (SESPA) Oviedo, Oviedo, Spain; 6 Hospital La Paz Institute for Health Research (IdiPAZ), Madrid, Spain; 7 Department of Psychiatry, Universidad Autónoma de Madrid (UAM), Madrid, Spain; 8 Hospital Clínico San Carlos, Madrid, Spain; 9 Department of Psychology, University of Oviedo, Oviedo, Spain; 10 Department of Mental Health, Corporació Sanitaria Parc Taulí de Sabadell, Barcelona, Spain; 11 Institut d’Investigació I Innovació Parc Taulí (I3PT), Institut de Neurociències, Unitat de Neurociència Traslacional, Parc Taulí Hospital Universitari, Universitat Autònoma de Barcelona, Bellaterra, Spain; 12 Department of Legal Medicine, Psychiatry and Pathology. School of Medicine, Complutense University of Madrid, Madrid, Spain; 13 Departament de Medicina, Facultat de Medicina I Ciències de La Salut, Universitat de Barcelona (UB), Barcelona, Spain; 14 Institut d’Investigacions Biomèdiques August Pi I Sunyer (IDIBAPS), Barcelona, Spain; 15 Department of Child and Adolescent Psychiatry and Psychology, Hospital Clinic of Barcelona, Barcelona, Spain; 16 Bipolar and Depressive Disorders Unit, Hospital Clinic de Barcelona, Barcelona, Spain; 17 Institute of Neurosciences (UBNeuro), Barcelona, Spain; 18 Department of Psychiatry, Clinical Psychology and Mental Health, La Paz University Hospital, Madrid, Spain; 19 Computer Science Department, University of Oviedo, Oviedo, Spain; 20 Computer Science Department, Munster Technological University, Rossa Avenue, Bishopstown, Cork Ireland; 21 Department of Psychiatry and Forensic Medicine, Autonomous University of Barcelona, Barcelona, Spain; 22 Institut Hospital del Mar d’Investigacions Mediques (IMIM), Barcelona, Spain; 23 BIOARABA. Hospital Universitario de Alava, University of the Basque Country, Leioa, Spain; 24 Department of Medicine & Psychiatry, University Hospital Virgen del Rocio, IBiS, Centro de Investigación Biomédica en Red de Salud Mental (CIBERSAM), Seville, Spain; 25 Facultad de Medicina, Departamento de Psiquiatría, Av. Julián Clavería, 6, 33006 Oviedo, Asturias Spain

**Keywords:** Adolescent, Suicide, Network analysis, Risk factors, Depression, Trauma

## Abstract

**Objective:**

This study aims to investigate the interrelationship of risk factors for suicidal behaviour and their influence on attempt severity in a sample of adolescents who have recently attempted suicide. For it a network analyse was performed.

**Method:**

Data from a sample of adolescents from 12 to 17 years of age with documented suicide attempts were collected and analysed in the context of a larger study conducted in Spain called SURVIVE. Several factors were examined including age, sex, depression, trauma, impulsivity, and substance abuse. Graph analysis was performed to identify relationships and centrality measures among these factors.

**Results:**

A total of 267 participants were enrolled: 233 females and 34 males with a mean age of 15.00 years (SD = 1.52). The results indicate that age and sex do not have a notable relationship with attempt severity in adolescents. Emotional and behavioural difficulties, measured by the Strengths and Difficulties Questionnaire (SDQ), have the greatest influence on other variables. Depression and childhood trauma show varying degrees of association with suicidal behaviour, as does motor impulsivity. Substance use does not appear to be strongly related to suicide attempt severity. The number of suicide attempts is strongly correlated with emotional and behavioural difficulties, depression, and childhood trauma. Lethality of suicide attempts and intensity of suicidal ideation do not show significant associations with the other variables.

**Conclusion:**

This study identifies significant risk factors for adolescent suicide. Emotional and behavioural symptoms, depression, and childhood trauma are strongly linked to suicidal behaviour. However, age, sex, and substance abuse show minimal correlation. Assessing emotional difficulties and depressive symptoms using specific questionnaires could be crucial in evaluating suicidal behaviour in adolescents.

**Supplementary Information:**

The online version contains supplementary material available at 10.1186/s13034-024-00842-9.

## Introduction

One of the major health and social challenges among young people is suicide. It is estimated that suicide is the third leading cause of death among individuals aged 15 to 19 worldwide [1]. In Spain, the rate has been gradually increasing over the past 10 years, and suicide became the leading cause of non-natural death among young people aged 14 to 19 in 2020 [2],. This problem affects not only the clinical population but also the general population, especially young people with certain risk factors [3].

Given the magnitude of these findings, primary and secondary suicide prevention is a crucial health objective [4], making it important to study and identify these risk factors that influence suicidal behaviour in adolescents.

Suicidal behaviour is a complex phenomenon that spans a broad spectrum of actions, ranging from suicidal ideation to death by suicide. Suicide attempts represent one of the most serious and extensively-researched behaviours [5]. Evaluating the severity of suicide attempts is crucial for assessing the level of risk and providing appropriate care [6]. This assessment encompasses multiple factors, such as the intensity of suicidal ideation, the method’s lethality, and the individual’s history of prior suicide attempts.

Risk factors for suicide in young people are multifaceted and include harmful alcohol use, childhood abuse, stigmatisation for seeking help, barriers to accessing care, and access to means of suicide [7].

One of the clinical risk factors that has been widely associated with suicide is depression. Although depression has been strongly linked to suicide in this age group [8, 9], it is not always present [10]. This leads us to believe that factors associated with suicide attempts are not solely clinical but have a wide origin.

Impulsivity is a factor that has been well studied and strongly linked to suicide in adolescence, especially among younger individuals [11–13]. Although impulsivity (mainly motor impulsivity) [14] is generally associated with suicide, the literature remains controversial. Some authors associate impulsivity and aggression only with female population [15, 16], while others do not associate impulsivity with suicide attempts or self-injurious behaviours [17].

Another extensively studied risk factor among adolescents who attempt suicide is alcohol and substance use. Most of the literature supports the idea that substance use is a risk factor for suicide attempts among both adolescents and young adults [16, 18, 19]. However, not all studies are consistent [20].

Furthermore, it has been observed that traumatic experiences and abuse during childhood contribute to the emergence of suicidality in adolescence [13, 16, 20, 21]. The influence can be direct or indirect through the role of mediators: post-traumatic stress disorder, depression, emotional dysregulation, impulsivity, low self-esteem, and dissociative symptoms [16, 21–23]. The diathesis-stress model asserts that stressful life events interact with vulnerability factors and increase the likelihood of suicidal behaviours [24]. Moreover, other situations perceived as particularly stressful by the child, such as migration or changing residence, legal problems, or the breakup of romantic relationships, may precede suicidal behaviour, likely through the development of adaptive problems [25]. Likewise, the severity of suicidal behaviour is related to exposure to stressful life events, these being more frequent in adolescents with suicidal behaviour than in those with ideation only [24, 26]. One of these stress factors is migration. There is limited literature on this topic, and the conclusions are inconsistent [27]. Recent systematic reviews support the idea that migration is a risk factor for suicide, with variations depending on country of birth and ethnicity [27, 28]. For example, young people of Latino or Asian origin who migrate to the United States show higher rates of suicidal ideation and suicide attempts, but not higher suicide death rates. Conversely, when comparing young people of the same ethnicity, those born in the United States have higher rates of ideation and suicide attempts [28]. These diverse results highlight the complexity of suicidal behaviour in relation to migration and cross-cultural differences. Part of this relationship is mediated through modulating factors such as socioeconomic status, limited access to social and healthcare resources, parent-child relationships, and duration of residency in the country [28, 29].

It is therefore necessary to continue studying the different risk factors for suicide in young populations in order to develop predictive models and prevention strategies.

Despite the extensive literature in this field, the contributions to clinical practice from the various studies conducted over the past 50 years are limited. There are numerous clinical guidelines whose effectiveness has not been adequately evaluated. A meta-analysis published in 2017 revealed that the predictive capacity of the most commonly studied risk factors is weak and inaccurate [30]. One of the main limitations when evaluating these factors is the methodological constraints of the existing studies. This does not imply that these risk factors lack relevance; rather, it highlights the need for different methodological approaches. The mechanisms by which these factors operate and the potential complex interrelations among them suggest that broader exploratory approaches could be interesting.

Within this context, the evidence found in a particularly vulnerable and under-researched risk group—adolescents—is even scarcer. It is known that the motivations behind suicide attempts vary by age and sex [31], making it essential to conduct specific studies within this population group.

Based on this need, we propose an exploratory network study that includes multiple risk factors gathered from a clinical adolescent population that has at least attempted suicide once. On one hand, we will relate these risk factors to the severity of the suicide attempt, and on the other hand, we will examine the relationships among the different factors. In this way, we aim to provide a broader and more complex view of the phenomenon of suicide.

### Aims of the study and hypotheses

In response to this need, we aim to conduct an exploratory network study that will analyze multiple risk factors among a clinical population of adolescents who have attempted suicide. This study will explore the relationship between these risk factors and the severity of the suicide attempt, as well as examine the interconnections among the various factors. To achieve this, several variables will be included such as sociodemographic data, history of migration and trauma, impulsivity, drug consumption, emotional distress, depression, and other mental health diagnoses.

Hypotheses:

We hypothesise that there are psychopathological and social factors that modulate the severity of suicidal attempts, which manifest during adolescence. These factors may be interrelated, and sex-related differences should be taken into account.

## Methods

### Study design and settings

This cross-sectional study is part of a larger nationwide project carried out in Spain, called SURVIVE (Suicide Prevention and Intervention) [32]. The SURVIVE project is a cohort study conducted by several research groups within the Spanish territory. It is led by the Hospital del Mar research group in Barcelona and is funded by the Carlos III Health Institute. The project had nine recruiting groups: Hospital Clinic (Barcelona), Corporació Sanitaria Parc Taulí (Barcelona), Hospital La Paz (Madrid), Hospital Clínico (Madrid), Hospital Central Universitario de Asturias (Oviedo), Hospital Santiago de Áraba, Hospital Virgen del Rocío (Seville), Hospital Río Hortega (Valladolid), and Hospital de Valdecilla (Santander). All the described centres belong to the public healthcare network managed within the public health system. They vary in size and capacity, ranging from approximately 500 beds to over 1,000 beds, and all of them are equipped to handle general medical emergencies as well as specialised emergency psychiatric care.

The recruitment period was from 1 January 2021 to 31 March 2023.

### Data collection and ethical considerations

Individuals who attempted suicide were screed by a psychiatrist at the emergency ward. After discharge, the participant was asked verbally, whether he/she consents to be called by a study team member to inform him/her about the study. If the patient consented, a study team member contacted the patient and the legal representative within the first 24 h to explain the study aims, and to arrange an appointment. Participants who agreed were given the Patient Information Sheet and Informed Consent. Participation is voluntarily and participants are allowed to abandon the study at any time. Adolescents were included in the study only if consent from both (the adolescent and his/her legal representative) is obtained. The adolescent’s consent was obtained through the provision of age-appropriate information. The project was evaluated at the Ethical Committees of each one of the participating sites.

In the initial assessment, only the adolescent and the evaluator were present, and it is conducted through an in-person interview in which data is collected and the patient completes the necessary questionnaires.

The current proposal complies with the national (code of ethics of the national board of medicine) and international (Declaration of Helsinki, Fortaleza, Brasil, October 2013) guidelines, and with the national regulations. Data protection will be assured following EU regulations.

### Sample

Baseline data from a national multisite cohort study conducted in individuals under 18 years old with a recent suicide attempt, recruited at nine university hospitals across Spain, were analysed as part of the “The Suicide Prevention and Intervention Study” (SURVIVE) [32].

#### Eligibility criteria

The study enrolled patients between the ages of 12 and 17 years who had been admitted to A&E for a suicide attempt in the 10 days prior to evaluation. It was required that both parents and participants be willing and able to comply with study procedures and provide written informed consent.

#### Exclusion criteria

Exclusion criteria included incapacity to give informed consent, lack of fluency in Spanish, and current participation in another clinical study that, in the investigator’s opinion, was likely to interfere with the objectives of the SURVIVE study.

#### Sociodemographic variables

During the assessment, demographic information was collected, including the participant’s age at the time of evaluation, the sex assigned at birth categorised as male or female based on visible external anatomy, and migration status whether they were born in Spain or not. Additionally, data regarding the current academic year in which the participant was enrolled, religious affiliation or beliefs, if any, and educational level of the parents or guardians were also recorded.

#### Clinical diagnosis

To assess the presence of psychiatric disorders, the Spanish version of the Mini International Neuropsychiatric Interview for Children and Adolescents (MINI-KID) [33] was used. The MINI-KID is a structured diagnostic interview designed for assessing various psychiatric disorders in children and adolescents. The interview consists of a series of questions based on criteria of the Diagnostic and Statistical Manual of Mental Disorders, 4th Edition (DSM-IV) and the International Statistical Classification of Diseases and Related Health Problems, 10th Revision (ICD-10). As the interviewer asks questions, they use a scoring system to determine if the child meets the criteria for specific disorders. After completing the interview, the clinician reviews the responses to make a final diagnostic decision.

Different diagnoses were grouped to facilitate the analysis as follows:

1. Affective disorders (including major depressive episode, dysthymic disorder and mood dysregulation disorder).
2. Anxiety disorders (panic disorder, agoraphobia, social anxiety, obsessive compulsive.disorder, post-traumatic stress disorder, and generalised anxiety disorder).
3. Alcohol-related disorders (alcohol abuse and alcohols dependence).
4. Drug-related disorders (drug abuse and drug dependence).
5. Attention deficit/hyperactivity disorder (ADHD).
6. Conduct disorders (conduct disorder and oppositional defiant disorder).
7. Psychotic disorders.
8. Eating disorders (anorexia nervosa and bulimia nervosa).
9. Adjustment disorders.

#### Depression

As previously mentioned in the introduction, depression is one of the risk factors most closely associated with suicide across all age groups [8, 9]. To conduct a more extended evaluation of depressive symptoms, a dual diagnostic approach has been proposed. On one hand, as previously noted, the MINI-KID scale was used. For this purpose, we included diagnoses related to depressive affective conditions, such as major depressive episode, dysthymic disorder and mood dysregulation disorder. On the other hand, we selected the ninth version of the PHQ scale [34] to assess subjective symptoms. The PHQ-9 is a brief, self-administered scale widely validated for assessing depressive symptoms. It has excellent internal consistency (Cronbach’s alpha > 0.8) and good sensitivity and specificity for detecting major depression. Higher scores suggest greater clinical severity of depressive symptoms, with a cut-off point set at 5.

This approach seems thorough, as it includes several diagnoses from the depressive spectrum and incorporates both subjective and objective assessments. Additionally, the PHQ scale is widely used in research, which can facilitate comparison with future studies.

#### Suicidal behaviour

To evaluate the severity of suicidal behaviour, the Columbia Suicide Severity Rating Scale (C-SSRS) [35] was administered. The C-SSRS is administered by the evaluator and has been extensively validated for its sensitivity and specificity in identifying individuals at risk for suicide. It has shown good interrater reliability and predictive validity for suicidal behavior.

For our research we have included the following outcomes:

- Lethality of the suicide attempt, measured as “actual lethality/medical damage”.
- Total number of attempts: first actual attempt vs. more than one attempt.
- Intensity of ideation (most severe type of ideation reported by the participant).

#### Drug consumption

The study was conducted using prior toxic substance use 2 h before the suicide attempt as a possible moderating variable. This included three types of substances: alcohol, cannabis, and other drugs (cocaine, heroin, and amphetamines). This information was collected by the interviewer at the time of the initial assessment, reported verbally by the patient himself.

#### Impulsiveness

Impulsiveness was measured using the Barratt Impulsiveness Scale Version 11 (BIS-11) [36]. It is a self-report questionnaire that has been validated across multiple populations and demonstrates solid internal consistency (Cronbach’s alpha ~ 0.80) and test-retest reliability for assessing impulsivity traits. This scale scores three subscales: attentional impulsiveness, motor impulsiveness, and non-planning impulsiveness. In this case, the total score was also included.

#### Trauma

History of traumatic events and child abuse was evaluated using the Childhood Trauma Questionnaire-Short Form (CTQ-SF) [37]. The CTQ-SF is a widely used instrument for assessing experiences of childhood abuse and neglect, with excellent internal consistency (Cronbach’s alpha > 0.90) and high test-retest reliability. It can be divided into five main subscales: emotional abuse, physical abuse, emotional neglect, physical neglect, and sexual abuse.

#### Emotional and behavioural difficulties

Through the Strengths and Difficulties Questionnaire (SDQ) [38], different emotional and behavioural aspects of adolescents have been assessed. The SDQ is a reliable and valid questionnaire that shows good internal consistency (alpha values around 0.73) and robust criterion validity. For the purpose of this study, five sub-scales were included: emotional symptoms, conduct problems, hyperactivity/inattention, peer problems, and prosocial behaviour, along with total score.

### Data analysis

A network analysis was conducted to examine the relationship between suicide risk factors, patient characteristics, and suicide attempt characteristics in our population. Networks consist of nodes and edges. In our case, nodes represent the variables of interest and edges represent the relationships between them.

To analyse the sociodemographic variables, descriptive statistics such as means and frequencies were used. Then, the relationship between the different variables was calculated using a Spearman correlation and a graph model (network) was built, where all the nodes (variables of interest) were connected to each other through edges. Each edge represents the Spearman correlation between two nodes. To facilitate data interpretation, we also built graphs excluding correlations below 0.2. This threshold was selected because a Spearman correlation coefficient of 0.2 is widely considered to indicate a *weak* correlation [39].

In the network, direct associations are represented by edges connecting two nodes, indicating a co-occurrence between the variables. Indirect associations arise when two nodes are not directly connected but are linked through intermediary nodes, suggesting that one variable may influence another via one or more mediating factors. It is important to emphasize that both direct and indirect associations represent correlations and should not be interpreted as causal relationships. Network analysis identifies patterns of association rather than establishing causal links. While the network reveals complex interrelationships among risk factors, these associations can generate hypotheses for further causal investigation but do not confirm causal pathways on their own.

Missing data were handled using an imputation approach, with mean imputation applied to numeric variables and mode imputation to categorical variables. The average proportion of missing values across the variables in the study was 1.27%.

The Fruchterman-Reingold [40] algorithm was used for visualisation of the relationships. Fruchterman-Reingold is a force-directed graph drawing algorithm for displaying graphs in two-dimensional space, in an aesthetically-pleasing and easy-to-understand way. Unconnected nodes repel one other and connected nodes attract one another. Then, after a number of iterations, a layout is achieved in which the distance between nodes corresponds to the absolute edge weight between those nodes. Thus closely related nodes are plotted near each other. The most influential nodes are centred on the graph, with the least influential ones in peripheral positions.

In graph theory and network analysis, node centrality measures assess the importance of a node in the network structure [], i.e. the variables that are more influential on the others in the network. The three following centrality measures were used:

- Strength centrality. Weighted networks are those for which a certain value is assigned to each of the edges. In this scenario, the weight of each is the correlation between the two nodes connected by the edge. Thus, the strength centrality of a node is the sum of the weight of its connections, in absolute value [42]. It represents the degree to which a variable can directly influence (or be influenced by) many other variables [43]. Strength centrality is particularly useful in understanding which risk factors have the most direct influence on other variables in the network. In our study, variables with high strength centrality exert a stronger influence on suicidal behaviour, highlighting key risk factors that are central in influencing suicidal behaviour, making them potential targets for intervention.
- Closeness centrality. The distance between two nodes is defined as the length of the shortest path between them [44]. Lengths are the inverse of the absolute weight, with highly correlated nodes connected by short paths and vice versa. Closeness centrality is defined as the inverse of the sum of the distances between one node and all other nodes in the network [45]. In this way, a closeness-centrality variable is one that is likely to be quickly affected by changes in another variable, directly or through changes in other variables. Thus, its influence can reach other variables more quickly than the influence of those that are peripheral in terms of closeness. In our study, a variable with high closeness centrality represents a factor that can rapidly spread its effects across the network. This measure is important because it suggests which factors might be the fastest to impact suicidal behaviour.
- Betweenness centrality. The geodesics between two nodes are the paths connecting them that have the shortest distance. Betweenness centrality is defined as the number of geodesics between any two nodes that pass through the focal one. Betweenness centrality assumes that the shortest paths are particularly important because they represent the strongest indirect correlation between two nodes [46]. A variable with high betweenness centrality serves as a bridge between otherwise weakly connected risk factors. For example, a factor like childhood trauma with high betweenness may act as a critical link between depression and emotional difficulties, even if these factors are not directly correlated. Identifying variables with high betweenness centrality can reveal important pathways influencing suicidal behaviour.

To assess the stability of edge and centrality measures, we employed the R bootnet package with non-parametric bootstrapping [47]. Specifically, 1000 samples were generated by resampling rows with replacement to estimate the stability of edge weights. For estimating the stability of centrality indices, we utilised the Correlation Stability coefficient (CS-coefficient) focusing on correlation values greater than or equal to *r* = 0.5 (CS-coefficient(*r* = 0.5)) [47]. The CS-coefficient indicates the percentage of our sample that can be dropped while still maintaining correlation values greater than or equal to *r* = 0.5, with a 95% confidence interval, between our sample’s centrality indices and those computed from our bootstrapped samples (figures in supplemental documentation).

This network analysis method is particularly effective for analysing the interrelationships among risk factors for suicidal behaviour and their influence on attempt severity. It allows us to explore the complex interactions among multiple risk factors in adolescents. By employing centrality measures, we can identify the most influential variables within the network, enhancing our understanding of how specific factors—such as emotional and behavioural difficulties, depression, and childhood trauma—are central to suicidal behaviour. This approach facilitates a nuanced examination of risk factor interactions that traditional statistical methods may overlook.

One of the key strengths of this network analysis method is its ability to visualize the interrelationships among risk factors for suicidal behaviour, providing a clear representation of how different risk factors are interconnected. This visual clarity aids in identifying potential targets for intervention and highlights the multifaceted nature of suicidal behaviour, which can inform more effective prevention strategies. Additionally, by focusing on the strength and direction of relationships between variables, this method allows for a more dynamic understanding of how risk factors may evolve and interact over time.

All the previously described centrality measures, together with the Fruchterman-Reingold algorithm, were computed using the R programming language and its qgraph 1.9.5 package for graph plotting and model estimation.

## Results

### Sociodemographic and clinical characteristics

Tables 1 and 2 present the sociodemographic and clinical characteristics of the study sample. The final sample included 267 patients, consisting of 233 females and 34 males, with a mean age of 15.00 years (SD = 1.52). The majority of patients (*n* = 216, 80.9%) were born in Spain and 51(19.1%) were born abroad. A total number of 191 (71.5%) were enrolled in compulsory secondary education at the time of the assessment.

**Table 1:** Sociodemographic characteristics of the sample (*n* = 267)

Variable	*N*	Mean	SD
Age	267	15.0	1.52
Variable	Category	N	Frequency (%)
Sex	Female	233	87.4
	Male	34	12.6
Home Country	Spain	216	80.9
	Europe	2	0.7
	Africa	1	0.4
	South America	23	8.6
	Asia	1	0.4
	Other	24	9.0
Religion	Yes	94	35.2
	No	163	61.0
	Others	7	2.6
	Not available	3	1.1
Current Academic Year	Primary Education	3	1.1
	Compulsory Secondary School	191	71.5
	Baccalaureate Degree	52	19.5
	Vocational Training	17	6.4
	Has Dropped out of School	4	1.5
Parental Education	No Education	4	1.5
	Primary School	16	6.0
	Secondary School	122	45.7
	University	98	36.7
	Does Not Know	27	10.1

**Table 2:** Clinical characteristic of the sample

Clinical variable	*N*	Mean	SD
Total number of attempts	267	2.54	2.39
PHQ 9 total score	265	17.97	5.58
Clinical variable	Categories	N	Frequency (%)
Intensity of the ideation	1 (least severe)	10	3.9
	2	12	4.7
	3	68	26.7
	4	67	26.3
	5 (most severe)	98	38.4
	Not available	12	4.5
Lethality of the attempt	No physical damage	56	21.0
	Minor physical damage	72	27.0
	Moderate physical damage	104	39.0
	Moderately severe physical damage	25	9.4
	Severe physical damage	10	3.7
Drug before attempt	Alcohol	18	6.8
	Cannabis	12	4.5
	Other drugs	1	0.4
Diagnosis (MINI-KID)	Affective disorder	183	68.5
	Anxiety disorders	119	44.5
	Alcohol-related disorder	7	2.6
	Drug-related disorder	9	3.4
	ADHD	23	8.6
	Conduct disorder	16	6.0
	Psychotic disorder	8	3.0
	Eating disorder	56	20.0
	Adjustment disorder	45	16.9
Depression (PHQ-9)	Clinical depression (total score > 5)	259	97.7
	Not available	2	

*PHQ-9* Patient Health Questionnaire, *MINI-KID* Mini International Neuropsychiatric Interview for Children and Adolescents, *ADHD* Attention Deficit Hyperactivity Disorder

Regarding variables associated with suicidal behaviour, the mean number of suicide attempts per patient was 2.54 (SD = 2.39), with a single suicide attempt being the most frequent value. In terms of ideation intensity, 38.9% (98 from 255) experienced their most severe suicidal ideation before the attempt (12 patients have no data).

Concerning lethality of the actual suicide attempt, 21.0% (*n* = 56) did not sustain any type of injury, 27.0% (*n* = 72) sustained minor injuries, 39.0% (*n* = 104) sustained moderate injuries, 9.4% (*n* = 25) sustained moderate injuries requiring hospitalisation, and 3.7% (*n* = 10) sustained severe injuries. Regarding substance use prior to the attempt, 6.7% (*n* = 18) of adolescents consumed alcohol, 4.5% (*n* = 12) used cannabis, and 0.4% (*n* = 1) used other drugs.

In terms of clinical diagnosis of the sample, the most frequently encountered issues were anxiety problems, followed by affective problems according to the MINI-KID. Based on PHQ-9 total scores, only 4 out of 265 patients (2 of which were missing PHQ test data) did not present with depressive symptoms (1.5%). The mean score of the sample was 17.97 (SD = 5.58), indicating moderately severe depression.

### Centrality measures

**Fig. 1:**
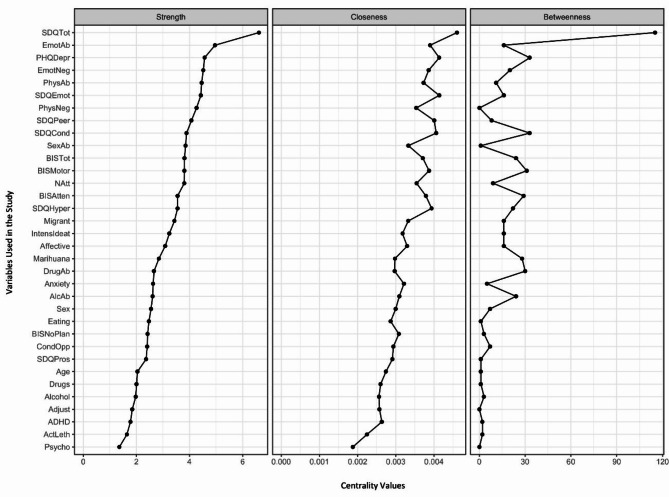
Strength, closeness, and betweenness centrality measures for all the variables, considering all the correlations. Note: Values of the Strength, Closeness, and Betweenness centrality measures for all the variables used in our study. Variables are ordered in a descending order based on their Strength value. The following variable abbreviations are used: *BIS* Barratt Impulsiveness Scale, *BISAtten* Attentional sub-score, *BISMotor* Motor sub-score, *BISNoPlan* Non-planning sub-score, *BISTot* Total score, *CTA* Characteristics of the Attempt, *Alcohol* consumption of alcohol prior to suicide attempt, *Marihuana* consumption of cannabis prior to suicide attempt, *Drugs* consumption of other drugs prior to suicide attempt, *CTQ* Childhood Trauma Questionnaire, *EmoAb* Emotional Abuse, *EmoNeg* Emotional Neglect, *PhysNeg* Physical Neglect, *SexAb* Sexual Abuse, *PhyAb* Physical Abuse, *SDQ* Strengths and Difficulties Questionnaire, *SDQEmot* Emotional symptoms, *SDQCond* Conduct problems, *SDQHyper* Hyperactivity/inattention, *SDQPeer* Peer problems, *SDQPros* Prosocial behaviour, *SDQTot* Total score, *MINI-KID* Mini International Neuropsychiatric Interview for Children and Adolescents, *Affective* Affective disorders, *Anxiety* Anxiety disorders, *ADHD* Attention deficit/hyperactivity disorder, *AlcAb* Alcohol-related disorder, *DrugAb* Drug-related disorder, *CondOpp* Conduct disorder and Oppositional defiant disorder, *Psycho* Psychotic disorder, *Eating* Anorexia and bulimia nervosa, *Adjust* Adjustment disorder, *PHQDep* Patient Health Questionnaire, *Natt* total number of suicide attempts, *IntensIdeat* intensity of suicidal thoughts, *ActLeth* Lethality of actual attempt, *Migrant* not born in Spain

**Fig. 2:**
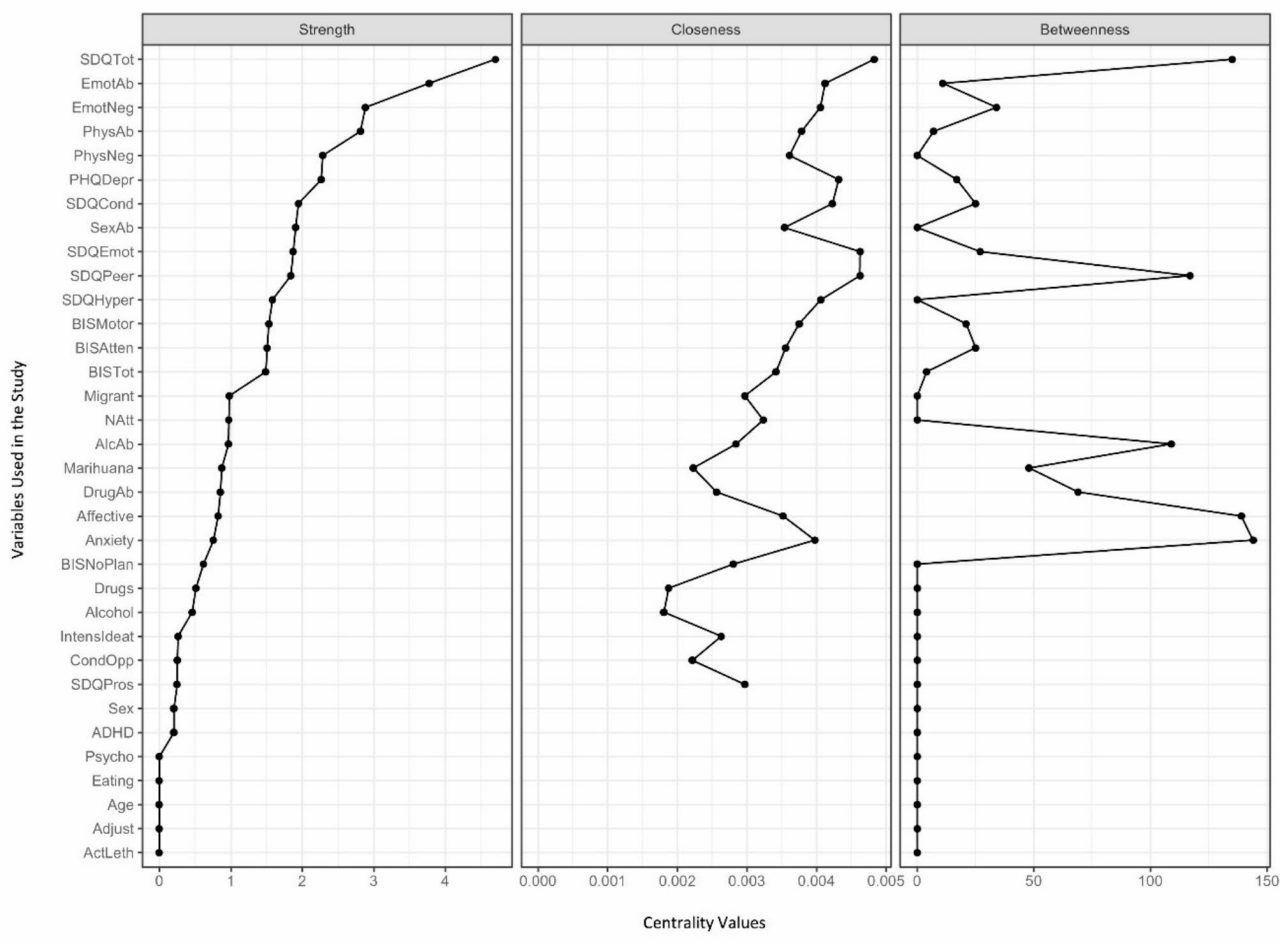
Strength, closeness, and betweenness centrality measures for all the variables, considering all the correlations with an absolute value of 0.2 or greater. Note: Values of the Strength, Closeness, and Betweenness centrality measures for all the variables used in our study, considering only those with a minimum correlation with absolute values greater than or equal to 0.2. Variables are ordered in a descending order based on their Strength value. The following variable abbreviations are used: *BIS* Barratt Impulsiveness Scale, *BISAtten* Attentional sub-score, *BISMotor* Motor sub-score, *BISNoPlan* Non-planning sub-score, *BISTot* Total score, *CTA* Characteristics of the Attempt, *Alcohol* consumption of alcohol prior to suicide attempt, *Marihuana* consumption of cannabis prior to suicide attempt, *Drugs* consumption of other drugs prior to suicide attempt, *CTQ* Childhood Trauma Questionnaire, *EmoAb* Emotional Abuse, *EmoNeg* Emotional Neglect, *PhysNeg* Physical Neglect, *SexAb* Sexual Abuse, *PhyAb* Physical Abuse, *SDQ* Strengths and Difficulties Questionnaire, *SDQEmot* Emotional symptoms, *SDQCond* Conduct problems, *SDQHyper* Hyperactivity/inattention, *SDQPeer* Peer problems, *SDQPros* Prosocial behaviour, *SDQTot* Total score, *MINI-KID* Mini International Neuropsychiatric Interview for Children and Adolescents, *Affective* Affective disorders, *Anxiety* Anxiety disorders, *ADHD* Attention deficit/hyperactivity disorder, *AlcAb* Alcohol-related disorder, *DrugAb* Drug-related disorder, *CondOpp* Conduct disorder and Oppositional defiant disorder, *Psycho* Psychotic disorder, *Eating* Anorexia and bulimia nervosa, *Adjust* Adjustment disorder, *PHQDep* Patient Health Questionnaire, *Natt* total number of suicide attempts, *IntensIdeat* intensity of suicidal ideas, *ActLeth* Lethality of actual attempt, Migrant: not born in Spain

Centrality measures are shown in Figs. 1 and 2, with Fig. 2 presenting the measures using a correlation threshold of 0.2. Across all cases, the total score on the SDQ scale emerged as the variable with the highest values in all three centrality measures. The high strength centrality indicates that the SDQ is strongly connected to many other risk factors, suggesting that the emotional and behavioural difficulties captured by the SDQ are directly linked to numerous other factors influencing suicidal behaviour and attempt severity. The SDQ plays a dominant and pervasive role in the network, directly influencing various other variables.

Another set of important variables are those related to emotional trauma, which ranked second in terms of strength centrality. This suggests that emotional trauma is strongly and directly connected to many other risk factors in the network, playing a similarly pervasive role in influencing suicidal behaviour and attempt severity among adolescents. These emotional trauma variables also ranked in the 85th percentile for closeness centrality, indicating that they are relatively central within the network and facilitate quicker interactions with other risk factors. Together, these findings highlight emotional trauma as a critical factor, both in its direct influence and in mediating the relationships between other risk factors.

The PHQ-9 variable, which assesses depressive symptoms, also shows a significantly high value in strength (ranking third among all correlations and sixth for those above 0.2) and closeness (88th percentile) centralities. These values indicate a strong direct connection with other factors related to suicidal behaviour and attempt severity among adolescents.

Variables such as age, sex, and lethality of the suicide attempt held less prominent positions, appearing at the lower end of the graphs for all three centrality measures.

Robustness results are included in supplemental documentation.

### Correlation between variables

**Fig. 3:**
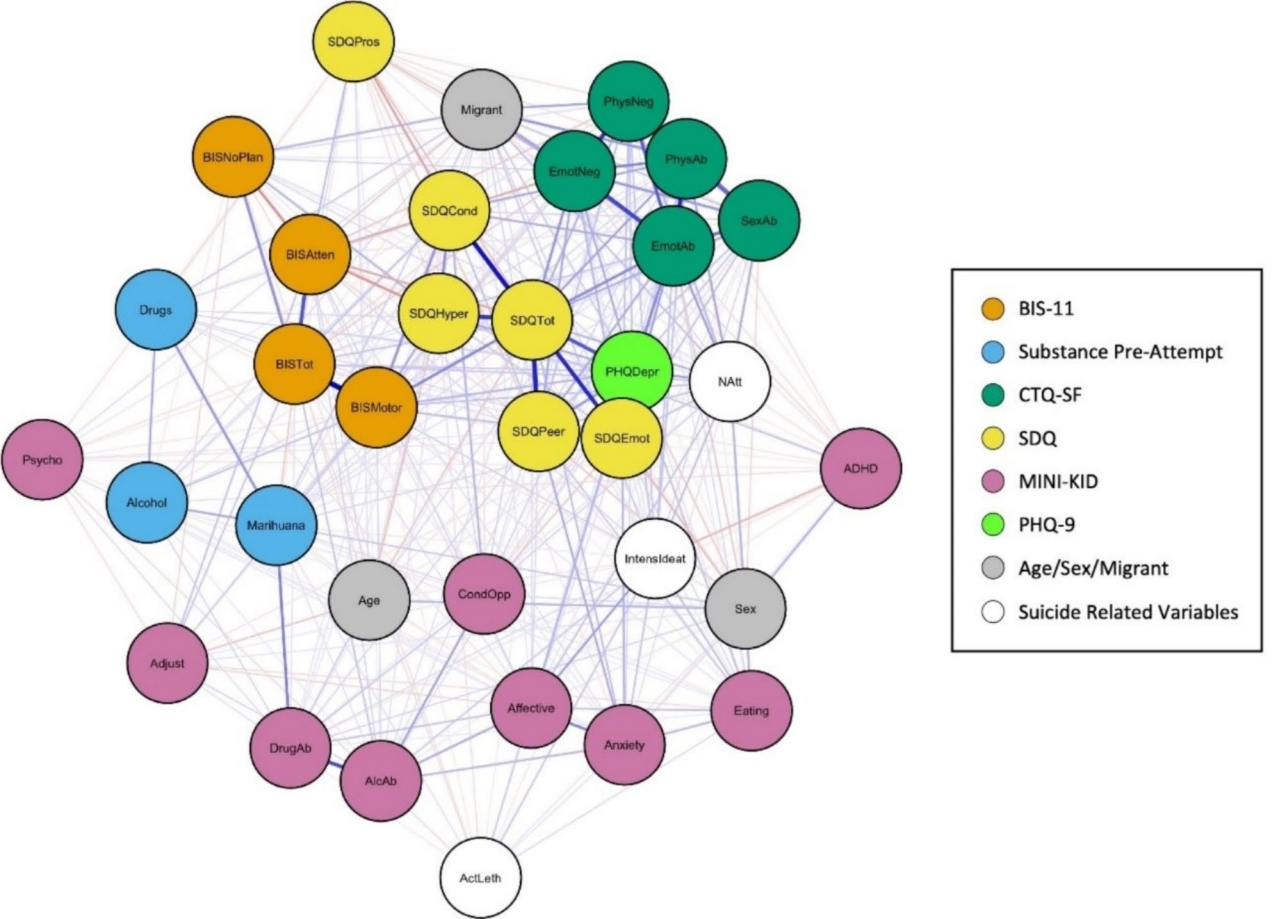
Fruchterman-Reingold graph representation of the spearman correlations. Note: Fruchterman-Reingold graph representation of the Spearman correlations between variables. Node colours represent the type of variable, edge width indicates the correlation value, blue edges represent positive correlations and orange edges showing negative correlations. The following variable abbreviations are used: *BIS* Barratt Impulsiveness Scale, *BISAtten* Attentional sub-score, *BISMotor* Motor sub-score, *BISNoPlan* Non-planning sub-score, *BISTot* Total score, *Alcohol* consumption of alcohol prior to suicide attempt, *Marihuana* consumption of cannabis prior to suicide attempt, *Drugs* consumption of other drugs prior to suicide attempt, *CTQ* Childhood Trauma Questionnaire, *EmoAb* Emotional Abuse, *EmoNeg* Emotional Neglect, *PhysNeg* Physical Neglect, *SexAb* Sexual Abuse, *PhyAb* Physical Abuse, *SDQ* Strengths and Difficulties Questionnaire, *SDQEmot* Emotional symptoms, *SDQCond* Conduct problems, *SDQHyper* Hyperactivity/inattention, *SDQPeer* Peer problems, *SDQPros* Prosocial behaviour, *SDQTot* Total score, *MINI-KID* Mini International Neuropsychiatric Interview for Children and Adolescents, *Affective* Affective disorders, *Anxiety* Anxiety disorders, *ADHD* Attention deficit/hyperactivity disorder, *AlcAb* Alcohol-related disorder, *DrugAb* Drug-related disorder, *CondOpp* Conduct disorder and Oppositional defiant disorder, *Psycho* Psychotic disorder, *Eating* Anorexia and bulimia nervosa, *Adjust* Adjustment disorder, *PHQDep* Patient Health Questionnaire, *Natt* total number of suicide attempts, *IntensIdeat* intensity of suicidal thoughts, *ActLeth* Lethality of actual attempt, Migrant: not born in Spain

**Fig. 4:**
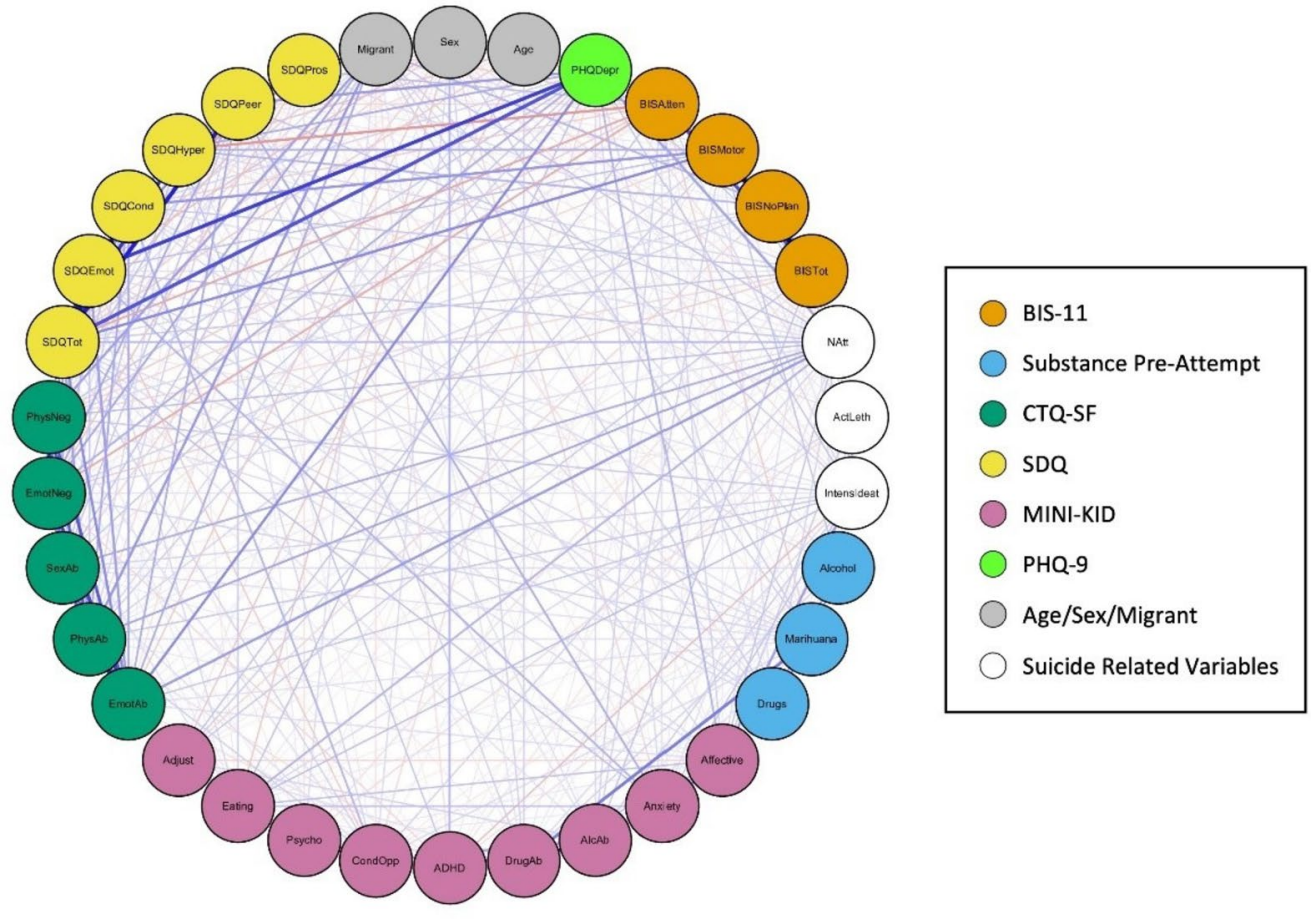
Graph (network) representation of the spearman correlations, placing all the nodes in a circle. Note: Graph (network) representation placing all the nodes in a circle. Node colours represent the type of variable, edge width indicates the correlation value, blue edges represent positive correlations and orange edges showing negative correlations. The following variable abbreviations are used: *BIS* Barratt Impulsiveness Scale, *BISAtten* Attentional sub-score, *BISMotor* Motor sub-score, *BISNoPlan* Non-planning sub-score, *BISTot* Total score, *CTA* Characteristics of the Attempt, *Alcohol* consumption of alcohol prior to suicide attempt, *Marihuana* consumption of cannabis prior to suicide attempt, *Drugs* consumption of other drugs prior to suicide attempt, *CTQ* Childhood Trauma Questionnaire, *EmoAb* Emotional Abuse, *EmoNeg* Emotional Neglect, *PhysNeg* Physical Neglect, *SexAb* Sexual Abuse, *PhyAb* Physical Abuse, *SDQ* Strengths and Difficulties Questionnaire, *SDQEmot* Emotional symptoms, *SDQCond* Conduct problems, *SDQHyper* Hyperactivity/inattention, *SDQPeer* Peer problems, *SDQPros* Prosocial behaviour, *SDQTot* Total score, *MINI-KID* Mini International Neuropsychiatric Interview for Children and Adolescents, *Affective* Affective disorders, *Anxiety* Anxiety disorders, *ADHD* Attention deficit/hyperactivity disorder, *AlcAb* Alcohol-related disorder, *DrugAb* Drug-related disorder, *CondOpp* Conduct disorder and Oppositional defiant disorder, *Psycho* Psychotic disorder, *Eating* Anorexia and bulimia nervosa, *Adjust* Adjustment disorder, *PHQDep* Patient Health Questionnaire, *Natt* total number of suicide attempts, *IntensIdeat* intensity of suicidal thoughts, *ActLeth* Lethality of actual attempt, Migrant: not born in Spain

Table 3 details the Spearman correlations between all the key variables included in the study. To represent this, Figs. 3 and 4 show two different visualisations of graphs (networks). Nodes (vertices) represent each variable and their colour represents their class (e.g. BIS-11, CTQ-SF, SDQ). Edge (link) widths are proportional to the correlation between variables, where positive correlation (direct relationship) is represented in blue, while negative correlation is represented in orange.

**Table 3:** Spearman correlations between all the key variables included in the study

	Sex	Age	PHQ	BISA	BISM	BISN	BIST	NAt	AcL	InI	Alc	Mrh	Drg	Aff	Anx	AlA	DrA
Sex	1.000	0.197	− 0.129	− 0.033	− 0.008	− 0.003	− 0.016	− 0.092	0.060	− 0.084	− 0.058	0.134	− 0.024	− 0.063	− 0.102	0.009	0.117
Age	0.197	1.000	− 0.017	0.107	− 0.018	− 0.097	0.002	− 0.044	0.002	− 0.062	0.014	0.054	− 0.045	0.021	− 0.046	0.030	0.125
PHQ	− 0.129	− 0.017	1.000	0.002	0.184	− 0.001	0.140	0.245	− 0.089	0.156	0.021	0.054	0.103	0.191	0.106	0.093	0.007
BISA	− 0.033	0.107	0.002	1.000	0.093	− 0.305	0.473	0.017	0.021	0.106	0.035	− 0.129	− 0.058	0.114	0.082	0.114	0.010
BISM	− 0.008	− 0.018	0.184	0.093	1.000	− 0.094	0.704	0.157	− 0.046	0.070	0.158	0.034	0.075	0.063	0.057	0.114	0.061
BISN	− 0.003	− 0.097	− 0.001	− 0.305	− 0.094	1.000	0.310	0.089	− 0.083	0.025	− 0.047	0.035	0.015	− 0.035	− 0.016	− 0.023	0.014
BIST	− 0.016	0.002	0.140	0.473	0.704	0.310	1.000	0.144	− 0.087	0.117	0.081	− 0.040	0.033	0.079	0.049	0.155	0.066
NAt	− 0.092	− 0.044	0.245	0.017	0.157	0.089	0.144	1.000	0.044	0.176	0.038	0.025	0.106	0.152	0.081	0.033	0.053
AcL	0.060	0.002	− 0.089	0.021	− 0.046	− 0.083	− 0.087	0.044	1.000	0.145	− 0.035	0.071	0.039	0.141	0.068	0.023	− 0.019
InI	− 0.084	− 0.062	0.156	0.106	0.070	0.025	0.117	0.176	0.145	1.000	0.037	− 0.054	− 0.064	0.261	0.159	0.132	0.095
Alc	− 0.058	0.014	0.021	0.035	0.158	− 0.047	0.081	0.038	− 0.035	0.037	1.000	0.230	0.228	0.050	− 0.052	0.049	0.113
Mrh	0.134	0.054	0.054	− 0.129	0.034	0.035	− 0.040	0.025	0.071	− 0.054	0.230	1.000	0.283	− 0.068	− 0.042	0.077	0.359
Drg	− 0.024	− 0.045	0.103	− 0.058	0.075	0.015	0.033	0.106	0.039	− 0.064	0.228	0.283	1.000	− 0.073	− 0.034	− 0.010	− 0.011
Aff	− 0.063	0.021	0.191	0.114	0.063	− 0.035	0.079	0.152	0.141	0.261	0.050	− 0.068	− 0.073	1.000	0.338	0.222	0.160
Anx	− 0.102	− 0.046	0.106	0.082	0.057	− 0.016	0.049	0.081	0.068	0.159	− 0.052	− 0.042	− 0.034	0.338	1.000	0.179	0.044
AlA	0.009	0.030	0.093	0.114	0.114	− 0.023	0.155	0.033	0.023	0.132	0.049	0.077	− 0.010	0.222	0.179	1.000	0.495
DrA	0.117	0.125	0.007	0.010	0.061	0.014	0.066	0.053	− 0.019	0.095	0.113	0.359	− 0.011	0.160	0.044	0.495	1.000
ADH	0.203	− 0.032	0.004	− 0.064	0.009	− 0.046	− 0.060	− 0.070	− 0.021	− 0.180	− 0.083	− 0.002	− 0.019	0.008	0.049	− 0.051	− 0.058
CnO	0.093	− 0.033	− 0.097	− 0.035	0.053	− 0.043	0.005	0.113	− 0.008	− 0.049	− 0.068	0.095	− 0.016	− 0.026	0.034	0.251	0.129
Psy	− 0.001	0.007	− 0.049	− 0.027	− 0.035	0.001	− 0.071	0.002	− 0.093	− 0.022	− 0.047	− 0.038	− 0.011	0.063	0.059	− 0.029	0.087
Etn	− 0.143	0.019	0.167	0.012	0.067	− 0.045	− 0.005	0.198	0.103	0.178	0.048	0.061	− 0.032	0.173	0.183	− 0.029	0.051
Adj	0.068	− 0.146	− 0.008	0.160	0.126	− 0.111	0.142	− 0.059	− 0.047	− 0.004	− 0.042	− 0.001	− 0.028	− 0.114	0.029	0.114	0.082
EmA	− 0.138	0.035	0.311	0.010	0.054	0.040	0.054	0.261	− 0.044	0.151	− 0.015	0.082	0.000	0.090	0.041	0.004	0.006
PhA	0.046	0.100	0.121	− 0.033	0.110	0.053	0.062	0.242	− 0.014	0.151	− 0.010	0.072	0.030	0.067	0.004	− 0.029	0.041
SxA	− 0.112	− 0.023	0.147	− 0.070	0.044	0.039	0.019	0.222	− 0.053	0.119	0.088	0.081	0.037	0.058	0.018	− 0.021	0.039
EmN	− 0.046	− 0.044	0.218	− 0.220	− 0.065	0.115	− 0.128	0.088	− 0.001	0.075	− 0.001	0.116	0.000	0.006	− 0.029	0.003	0.040
PhN	− 0.026	0.033	0.148	− 0.137	− 0.104	0.098	− 0.099	0.180	0.017	0.145	0.004	0.086	0.020	0.020	0.001	− 0.013	0.042
SDQT	− 0.113	− 0.140	0.470	− 0.231	0.317	0.106	0.151	0.192	− 0.038	0.067	0.032	0.093	0.105	0.093	0.173	0.073	0.051
SDQE	− 0.173	− 0.053	0.526	− 0.043	0.176	− 0.023	0.082	0.155	0.019	0.076	0.014	0.110	0.100	0.091	0.201	− 0.010	0.008
SDQC	− 0.015	− 0.081	0.118	− 0.128	0.288	0.037	0.155	0.083	0.039	0.032	0.094	0.082	0.101	0.036	− 0.002	− 0.001	− 0.021
SDQH	0.041	− 0.160	0.223	− 0.277	0.222	0.194	0.105	0.065	− 0.083	0.034	0.051	0.025	− 0.003	− 0.005	− 0.025	0.075	0.055
SDQPer	− 0.134	− 0.059	0.271	− 0.103	0.067	0.055	0.025	0.161	− 0.047	0.014	− 0.016	0.029	0.089	0.106	0.217	0.079	0.116
SDQPrs	− 0.055	0.086	− 0.022	0.177	0.050	− 0.028	0.098	0.050	− 0.035	0.066	0.017	− 0.058	− 0.081	0.035	0.064	0.048	− 0.015
Mgr	− 0.014	0.104	0.128	− 0.124	− 0.086	0.192	− 0.049	0.163	0.005	0.130	0.097	0.125	0.128	0.061	0.044	− 0.019	0.070
	ADH	CnO	Psy	Etn	Adj	EmA	PhA	SxA	EmN	PhN	SDQT	SDQE	SDQC	SDQH	SDQPer	SDQPrs	Mgr
Sex	0.203	0.093	− 0.001	− 0.143	0.068	− 0.138	0.046	− 0.112	− 0.046	− 0.026	− 0.113	− 0.173	− 0.015	0.041	− 0.134	− 0.055	− 0.014
Age	− 0.032	− 0.033	0.007	0.019	− 0.146	0.035	0.100	− 0.023	− 0.044	0.033	− 0.140	− 0.053	− 0.081	− 0.160	− 0.059	0.086	0.104
PHQ	0.004	− 0.097	− 0.049	0.167	− 0.008	0.311	0.121	0.147	0.218	0.148	0.470	0.526	0.118	0.223	0.271	− 0.022	0.128
BISA	− 0.064	− 0.035	− 0.027	0.012	0.160	0.010	− 0.033	− 0.070	− 0.220	− 0.137	− 0.231	− 0.043	− 0.128	− 0.277	− 0.103	0.177	− 0.124
BISM	0.009	0.053	− 0.035	0.067	0.126	0.054	0.110	0.044	− 0.065	− 0.104	0.317	0.176	0.288	0.222	0.067	0.050	− 0.086
BISN	− 0.046	− 0.043	0.001	− 0.045	− 0.111	0.040	0.053	0.039	0.115	0.098	0.106	− 0.023	0.037	0.194	0.055	− 0.028	0.192
BIST	− 0.060	0.005	− 0.071	− 0.005	0.142	0.054	0.062	0.019	− 0.128	− 0.099	0.151	0.082	0.155	0.105	0.025	0.098	− 0.049
NAt	− 0.070	0.113	0.002	0.198	− 0.059	0.261	0.242	0.222	0.088	0.180	0.192	0.155	0.083	0.065	0.161	0.050	0.163
AcL	− 0.021	− 0.008	− 0.093	0.103	− 0.047	− 0.044	− 0.014	− 0.053	− 0.001	0.017	− 0.038	0.019	0.039	− 0.083	− 0.047	− 0.035	0.005
InI	− 0.180	− 0.049	− 0.022	0.178	− 0.004	0.151	0.151	0.119	0.075	0.145	0.067	0.076	0.032	0.034	0.014	0.066	0.130
Alc	− 0.083	− 0.068	− 0.047	0.048	− 0.042	− 0.015	− 0.010	0.088	− 0.001	0.004	0.032	0.014	0.094	0.051	− 0.016	0.017	0.097
Mrh	− 0.002	0.095	− 0.038	0.061	− 0.001	0.082	0.072	0.081	0.116	0.086	0.093	0.110	0.082	0.025	0.029	− 0.058	0.125
Drg	− 0.019	− 0.016	− 0.011	− 0.032	− 0.028	0.000	0.030	0.037	0.000	0.020	0.105	0.100	0.101	− 0.003	0.089	− 0.081	0.128
Aff	0.008	− 0.026	0.063	0.173	− 0.114	0.090	0.067	0.058	0.006	0.020	0.093	0.091	0.036	− 0.005	0.106	0.035	0.061
Anx	0.049	0.034	0.059	0.183	0.029	0.041	0.004	0.018	− 0.029	0.001	0.173	0.201	− 0.002	− 0.025	0.217	− 0.064	0.044
AlA	− 0.051	0.251	− 0.029	− 0.029	0.114	0.004	− 0.029	− 0.021	0.003	− 0.013	0.073	− 0.010	− 0.001	0.075	0.079	0.048	− 0.019
DrA	− 0.058	0.129	0.087	0.051	0.082	0.006	0.041	0.039	0.040	0.042	0.051	0.008	− 0.021	0.055	0.116	− 0.015	0.070
ADH	1.000	− 0.022	− 0.054	− 0.031	0.040	− 0.080	− 0.081	− 0.021	− 0.102	− 0.046	0.014	− 0.035	0.012	0.151	− 0.045	0.002	− 0.080
CnO	− 0.022	1.000	− 0.045	− 0.054	0.052	0.048	0.120	0.082	0.015	0.080	0.118	− 0.135	0.193	0.111	0.054	0.045	− 0.082
Psy	− 0.054	− 0.045	1.000	− 0.027	− 0.079	− 0.051	0.008	0.030	− 0.029	0.050	− 0.013	− 0.065	− 0.011	0.029	0.059	− 0.079	0.084
Etn	− 0.031	− 0.054	− 0.027	1.000	− 0.089	0.109	− 0.011	0.038	0.081	0.064	0.059	0.102	− 0.029	− 0.065	0.140	0.013	− 0.041
Adj	0.040	0.052	− 0.079	− 0.089	1.000	0.010	0.031	0.025	− 0.067	− 0.029	0.028	− 0.003	0.010	0.027	0.001	− 0.033	− 0.037
EmA	− 0.080	0.048	− 0.051	0.109	0.010	1.000	0.599	0.402	0.516	0.456	0.311	0.259	0.219	0.051	0.218	− 0.077	0.216
PhA	− 0.081	0.120	0.008	− 0.011	0.031	0.599	1.000	0.425	0.340	0.448	0.252	0.150	0.227	0.095	0.113	− 0.090	0.277
SxA	− 0.021	0.082	0.030	0.038	0.025	0.402	0.425	1.000	0.272	0.361	0.223	0.159	0.116	0.149	0.151	− 0.055	0.148
EmN	− 0.102	0.015	− 0.029	0.081	− 0.067	0.516	0.340	0.272	1.000	0.566	0.284	0.188	0.167	0.075	0.231	− 0.152	0.234
PhN	− 0.046	0.080	0.050	0.064	− 0.029	0.456	0.448	0.361	0.566	1.000	0.203	0.160	0.073	0.045	0.168	− 0.089	0.250
SDQT	0.014	0.118	− 0.013	0.059	0.028	0.311	0.252	0.223	0.284	0.203	1.000	0.594	0.651	0.549	0.610	− 0.164	0.088
SDQE	− 0.035	− 0.135	− 0.065	0.102	− 0.003	0.259	0.150	0.159	0.188	0.160	0.594	1.000	0.130	0.137	0.292	0.040	0.114
SDQC	0.012	0.193	− 0.011	− 0.029	0.010	0.219	0.227	0.116	0.167	0.073	0.651	0.130	1.000	0.310	0.158	− 0.249	− 0.019
SDQH	0.151	0.111	0.029	− 0.065	0.027	0.051	0.095	0.149	0.075	0.045	0.549	0.137	0.310	1.000	0.001	− 0.086	0.023
SDQPer	− 0.045	0.054	0.059	0.140	0.001	0.218	0.113	0.151	0.231	0.168	0.610	0.292	0.158	0.001	1.000	− 0.126	0.115
SDQPrs	0.002	0.045	− 0.079	0.013	− 0.033	− 0.077	− 0.090	− 0.055	− 0.152	− 0.089	− 0.164	0.040	− 0.249	− 0.086	− 0.126	1.000	− 0.084
Mgr	− 0.080	− 0.082	0.084	− 0.041	− 0.037	0.216	0.277	0.148	0.234	0.250	0.088	0.114	− 0.019	0.023	0.115	− 0.084	1.000

*BIS* Barratt Impulsiveness Scale, *BISA* Attentional sub-score, *BISM* Motor sub-score, *BISN* Non-planning sub-score, *BIST* Total score, *Alc* consumption of alcohol prior to suicide attempt, *Mrh* consumption of cannabis prior to suicide attempt, *Drg* consumption of other drugs prior to suicide attempt, *CTQ* Childhood Trauma Questionnaire, *EmA* Emotional Abuse, *EmN* Emotional Neglect, *PhN* Physical Neglect, *SxA* Sexual Abuse, *PhA* Physical Abuse, *SDQ* Strengths and Difficulties Questionnaire, *SDQE* Emotional symptoms, *SDQC* Conduct problems, *SDQH* Hyperactivity/inattention, *SDQPer* Peer problems, *SDQPrs* Prosocial behaviour, *SDQT* Total score, *MINI-KID* Mini International Neuropsychiatric Interview for Children and Adolescents, *Aff* Affective disorders, *Anx* Anxiety Disorders, *ADH* Attention deficit/hyperactivity disorder, *AlcA* Alcohol-related disorder, *DrgA* Drug-related disorder, *CnO* Conduct disorder and Oppositional defiant disorder, *Psy* Psychotic disorder, *Etn* Anorexia and bulimia nervosa, *Adj* Adjustment disorder, *PHQ* Patient Health Questionnaire, *NAt* total number of suicide attempts, *InI* intensity of suicidal thoughts, *ActL* Lethality of actual attempt, *Mgr* Migration

**Fig. 5:**
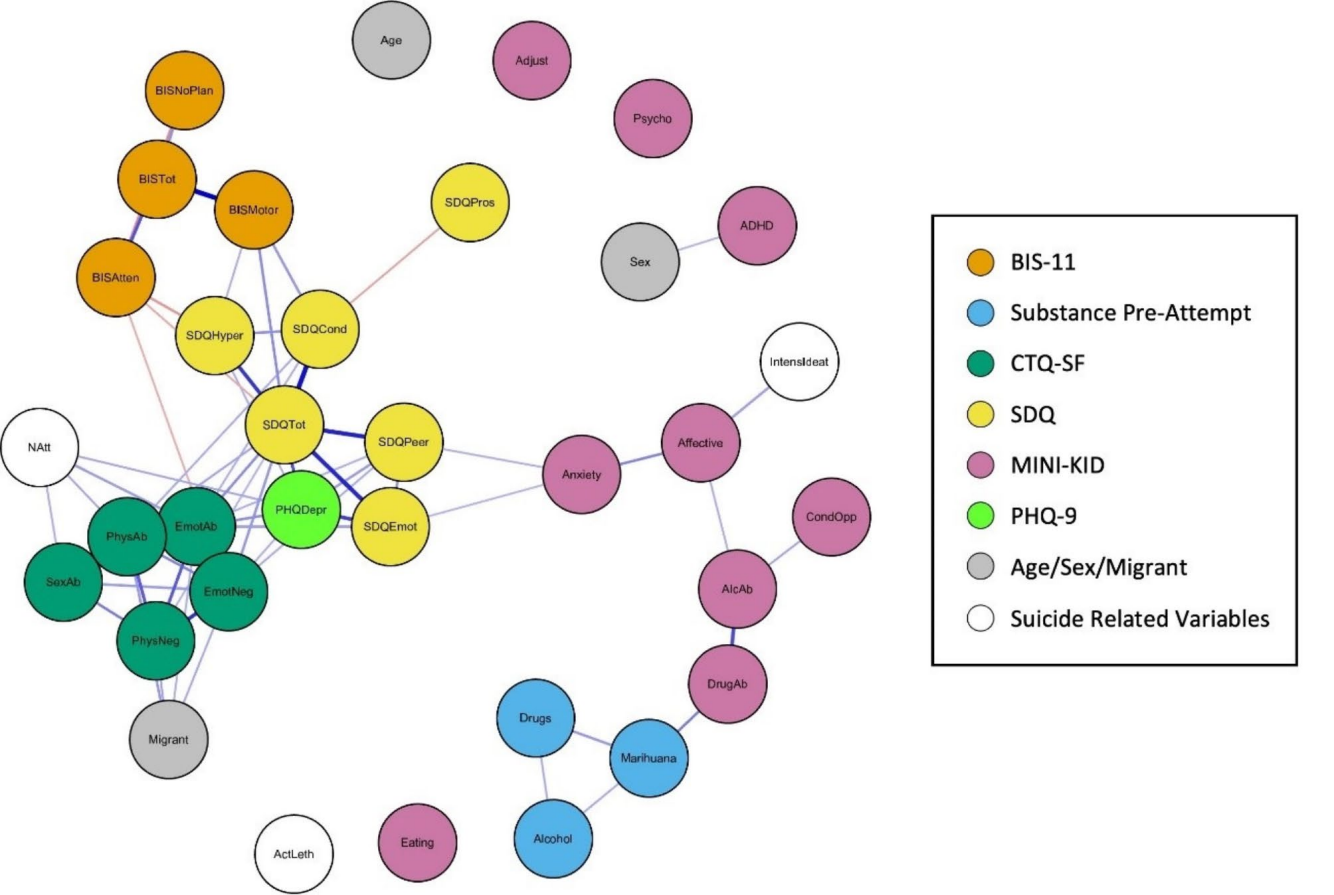
Fruchterman-Reingold graph representation of the spearman correlations with an absolute value of 0.2 or greater. Note. Fruchterman-Reingold graph representation of the Spearman correlations between variables, considering only those with absolute values greater than or equal to 0.2. Node colours represent the type of variable, edge width indicates the correlation value, blue edges represent positive correlations and orange edges showing negative correlations. The following variable abbreviations are used: *BIS* Barratt Impulsiveness Scale, *BISAtten* Attentional sub-score, *BISMotor* Motor sub-score, *BISNoPlan* Non-planning sub-score, *BISTot* Total score, *CTA* Characteristics of the Attempt, *Alcohol* consumption of alcohol prior to suicide attempt, *Marihuana* consumption of cannabis prior to suicide attempt, *Drugs* consumption of other drugs prior to suicide attempt, *CTQ* Childhood Trauma Questionnaire, *EmoAb* Emotional Abuse, *EmoNeg* Emotional Neglect, *PhysNeg* Physical Neglect, *SexAb* Sexual Abuse, *PhyAb* Physical Abuse, *SDQ* Strengths and Difficulties Questionnaire, *SDQEmot* Emotional symptoms, *SDQCond* Conduct problems, *SDQHyper* Hyperactivity/inattention, *SDQPeer* Peer problems, *SDQPros* Prosocial behaviour, *SDQTot* Total score, *MINI-KID* Mini International Neuropsychiatric Interview for Children and Adolescents, *Affective* Affective disorders, *Anxiety* Anxiety disorders, *ADHD* Attention deficit/hyperactivity disorder, *AlcAb* Alcohol-related disorder, *DrugAb* Drug-related disorder, *CondOpp* Conduct disorder and Oppositional defiant disorder, *Psycho* Psychotic disorder, *Eating* Anorexia and bulimia nervosa, *Adjust* Adjustment disorder, *PHQDep* Patient Health Questionnaire, *Natt* total number of suicide attempts, *IntensIdeat* intensity of suicidal thoughts, *ActLeth* Lethality of actual attempt, Migrant: not born in Spain

The graph in Fig. 3 was drawn with the Fruchterman-Reingold algorithm [40]. Figure 5 shows another graph drawn with the Fruchterman-Reingold algorithm but including only those with at least a weak correlation (those whose absolute values were greater than or equal to 0.2) [39]. Both figures visually support the previously mentioned centrality values. Graphs in Figs. 3 and 5 prominently display the SDQ total score variable at the centre, indicating the strongest correlations through wider edges and blue colouring, thereby reaffirming its crucial role in the network of risk factors. The SDQ variables are shown to have the most substantial influence within the network. Additionally, variables related to emotional trauma and depressive symptoms exert significant influence on the emotional and behavioural difficulties captured by the SDQ, forming the closest group of related variables. Consistent with the centrality measures, variables such as age, sex, and lethality of the suicide attempt are not centrally located in the graphs.

**Fig. 6:**
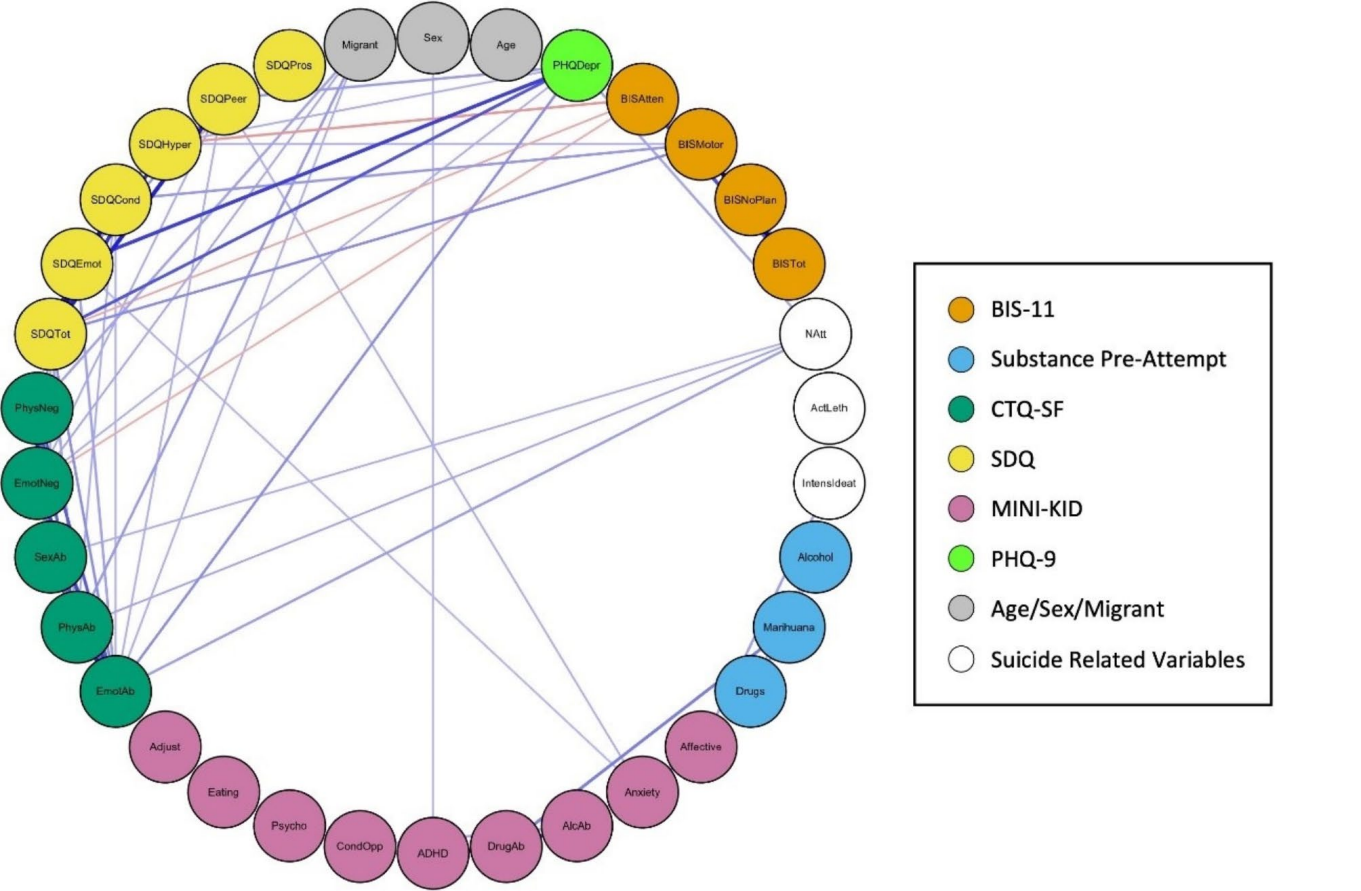
Graph (network) representation of the spearman correlations with an absolute value of 0.2 or greater, placing all the nodes in a circle. Note: Graph (network) representation placing all the nodes in a circle, considering only correlations with absolute values greater than or equal to 0.2. Node colours represent the type of variable, edge width indicates the correlation value, blue edges represent positive correlations and orange edges showing negative correlations. The following variable abbreviations are used: *BIS* Barratt Impulsiveness Scale, *BISAtten* Attentional sub-score, *BISMotor* Motor sub-score, *BISNoPlan* Non-planning sub-score, *BISTot* Total score, *Alcohol* consumption of alcohol prior to suicide attempt, *Marihuana* consumption of cannabis prior to suicide attempt, *Drugs* consumption of other drugs prior to suicide attempt, *CTQ* Childhood Trauma Questionnaire, *EmoAb* Emotional Abuse, *EmoNeg* Emotional Neglect, *PhysNeg* Physical Neglect, *SexAb* Sexual Abuse, *PhyAb* Physical Abuse, *SDQ* Strengths and Difficulties Questionnaire, *SDQEmot* Emotional symptoms, *SDQCond* Conduct problems, *SDQHyper* Hyperactivity/inattention, *SDQPeer* Peer problems, *SDQPros* Prosocial behaviour, *SDQTot* Total score, *MINI-KID* Mini International Neuropsychiatric Interview for Children and Adolescents, *Affective* Affective disorders, *Anxiety* Anxiety disorders, *ADHD* Attention deficit/hyperactivity disorder, *AlcAb* Alcohol-related disorder, *DrugAb* Drug-related disorder, *CondOpp* Conduct disorder and Oppositional defiant disorder, *Psycho* Psychotic disorder, *Eating* Anorexia and bulimia nervosa, *Adjust* Adjustment disorder, *PHQDep* Patient Health Questionnaire, *Natt* total number of suicide attempts, *IntensIdeat* intensity of suicidal thoughts, *ActLeth* Lethality of actual attempt

Figure 4 presents the correlation graph with all nodes arranged in a circle, while Fig. 6 filters this information to display only correlations of 0.2 or higher. This layout offers a clear and organized visual representation of the relationships between variables, minimizing visual clutter and complexity. The circular arrangement facilitates the easy interpretation of correlations, allowing key relationships between variables to be revealed more intuitively, without the level of detail presented in Table 3.

## Discussion

This cross-sectional study was conducted to assess the relationship between suicidal behaviour and various risk factors present in adolescents who have attempted suicide at least once. To facilitate interpretation of the results, only relationships whose absolute values were greater than or equal to 0.2 are taken into account [39].

### Sociodemographic risk factors

Regarding age and sex, the quantity and intensity of the relationships established were lower than expected. Their positions are very peripheral, and correlations are scarce: age is not related to any other variable, and sex is related only to ADHD (male sex).

Contrary to the existing literature [48], there is no relationship between female sex and higher number of suicide attempts or greater intensity of suicidal ideation. Similarly, male sex did not exhibit greater lethality in suicide attempts. Regarding age, there was also no direct or inverse relationship with variables associated with suicide.

More recent studies and reviews have found that the influence of sex on suicidal behaviour in adolescents is age-dependent [49]. While there are sex differences in early and mid-adolescence, these differences tend to diminish as individuals grow older and become approximately equal by the age of 19 [50]. According to this study, the greatest differences between boys and girls regarding suicide attempts occur between the ages of 15 and 16. Given that the average age in our sample is 15, significant differences should be evident, but they are not observed. Several studies conducted among college students have been unable to establish significant sex-related differences, but the population in this case is older [49].

Our results may seem contradictory, but it is important to note that the majority of the sample consists of girls. It would be interesting to replicate the study in a sample with greater male representation to see if the expected differences in adolescence are revealed.

### Migration

Migration appears to be a significant yet understudied risk factor for suicide [27]. Our findings indicate that, although it does not have a direct connection with suicide severity variables, it exhibits a more centralised distribution and is associated with trauma-related variables. Previous research indicates that mental well-being of migrants can be significantly impacted by pre-migratory circumstances, including traumas endured prior to and during the migration process [51].

Another noteworthy fact is the percentage of adolescents in the sample who have a history of migration. According to Spanish national statistics for the year 2022 [52], 10.2% of the population in Spain aged 12 to 17 years were born abroad, which is nearly half the percentage found in this sample. Despite the need for further in-depth analysis, this overrepresentation of migrants in our sample of suicide attempters highlights the vulnerability for suicide of this population.

### Emotional and behavioural difficulties

The SDQ variables exert the most significant influence on other variables and occupy central positions on the graph. Despite not being directly connected to suicide variables, the strong correlation between SDQ variables, especially emotional symptoms, and the PHQ-9 is of particular interest. This indirect association extends to the total number of suicide attempts.

The SDQ’s high closeness centrality signifies that it is more quickly reachable from other risk factors (i.e., through fewer steps) compared to other variables, functioning as a central hub in the network. This suggests that emotional and behavioural difficulties, as measured by the SDQ, act as key mediators or facilitators of other risk factors, enabling their influence to spread rapidly across the network. This central role underlines the SDQ’s critical importance within the overall structure of risk factors for suicidal behaviour.

Additionally, the SDQ’s high betweenness centrality indicates that it serves as a key connector or broker between other risk factors. Emotional and behavioural difficulties captured by the SDQ often mediate the relationships between other variables, playing an essential role in linking otherwise less connected factors. Therefore, the SDQ is significant not only for its direct impact but also for its ability to bridge various risk factors in the network.

The fact that the SDQ ranks highest across all three centrality measures (strength, closeness, and betweenness) underscores its pivotal role in the network of risk factors. Addressing the difficulties identified by the SDQ could potentially disrupt the broader risk factor network, thereby reducing the overall risk of suicide attempts. Interventions targeting these behavioural and emotional issues could have a significant ripple effect in mitigating suicide risk, given their central role in the network.

In the literature, there are few studies that link suicide to emotional and behavioural difficulties measured with the SDQ. There is one study conducted in 134 adolescents that focused on quality of life and suicide. It associates the SDQ emotional problems scale, SDQ peer problems scale, and SDQ total difficulties scale with a higher risk of suicide, especially in older adolescents [53]. Another study conducted in Korea found a relationship between the SDQ scale and increased suicidal behaviour in students, although it was based only on teacher’s reports [54].

On the other hand, the association between self-report measures and suicidality has been suggested in several studies, indicating that these questionnaires may be reliable and useful for evaluating suicide risk.

Regarding adolescents, specifically clinical population, studies indicate that self-report measures have been shown to be predictors of both suicidality and suicide attempts [55], not only in inpatients but also in Emergency Room [56].

The feasibility of using self-report measures in various settings, including schools and clinics, may make them advantageous for widespread screening. Their accessibility could contribute to an efficient assessment of suicide risk in diverse populations, facilitating timely interventions. Also, could empower adolescents to voice their experiences, fostering a supportive environment for discussing mental health issues.

The findings in the present study open a window of opportunities to study validated assessment quetionnaires in adolescents that may have significance when assessing suicide risk.

However, it’s important to recognize that such evaluations should not solely rely on diagnostic tools, as assessing suicide risk involves considering a broader range of factors.

### Depression

In this study, depressive symptoms were assessed with both the PHQ-9 and the MINI-KID scale. The PHQ-9 shows stronger influence and centrality compared to the MINI-KID depression variable, establishing a greater relationship with SDQ variables, CTQ-SF variables, and total number of suicide attempts. Regarding the diagnosis of depression with the MINI-KID, despite being directly related to intensity of ideation, it occupies a more peripheral position on the graph and is less strongly associated with other variables. However, isolated links between anxiety disorders and alcohol abuse can be observed.

It is worth noting that the relationship between the PHQ-9 variable and depression assessed with the MINI-KID is very weak (and non-existent at a correlation threshold of 0.2), despite the fact that both measure similar items. No articles were found that compare or relate the PHQ questionnaire and the MINI-KID in assessing suicide risk or depressive symptoms in adolescents. Here we hypothesize about the possible causes of these findings.

On one hand, the PHQ-9 is a brief, self-administered questionnaire, which may make adolescents feel more comfortable reporting their symptoms. This accessibility can lead to greater honesty in their responses, potentially resulting in better detection of depression. Additionally, the PHQ-9 evaluates depressive symptoms within a two-week timeframe, which may be more relevant for some adolescents who might not recognize or recall more chronic depressive symptoms that the MINI-KID aims to identify. It is also important to consider that the patients have recently attempted suicide and are experiencing a higher level of distress in the previous days.

Furthermore, the validity of each tool may vary depending on the specific population being assessed. In this case, the study sample includes a higher proportion of female adolescents, who are generally more likely to recognize and express their emotions [57]. This may explain why the PHQ-9 could more accurately reflect their subjective experiences.

Finally, the conditions under which the questionnaire are administered, as well as the relationship between the evaluator and the evaluated, can influence the results. If adolescents feel more comfortable with the approach of the PHQ-9, this may translate into more accurate responses.

A Spanish literature review identified psychological factors (depression, anxiety, previous suicide attempts, drug and alcohol use, and other comorbid psychiatric disorders), life stressors (family problems and peer conflicts), and personality traits (neuroticism and impulsivity) as factors that can increase the risk of suicidal behaviour [25]. Supporting the multifactorial origin of suicidal behaviour, a study comparing suicidal behaviour in adolescent Swedish twins found that suicide attempts were related to childhood psychopathology in a general sense, as well as genetic factors. However, when studying specific individual factors such as inattention, opposition, and anxiety, no statistically significant relationship was found with suicide attempts or self-injurious behaviours [17].

Based on our results, this study aligns with some of those previously discussed the literature, in which depression emerges as one of the most significant risk factors [8, 9]. Furthermore, when assessing depressive symptoms in patients with a recent suicide attempt, it may be advisable to use the PHQ-9 questionnaire rather than the MINI-KID.

### Childhood trauma

As expected, all the subscales of the CTQ-SF have a centralised position, closely related to SDQ variables, PHQ-9, and migrant situation and are also closely related to each other. Subscales for emotional abuse, sexual abuse, and physical abuse also are strongly associated with the number of previous suicide attempts.

A review of studies that relate suicide to different types of reported trauma found that extensive literature exists. In a study conducted in Brazil with a cohort of over a thousand young individuals, a decreasing magnitude of risk was observed for emotional abuse, emotional neglect, sexual abuse, physical abuse, and physical neglect [58]. In European [59] and clinical populations, a higher risk was found for emotional abuse than the other types of trauma [60, 61].

In this cohort, patients with a history of migration are most closely associated with physical abuse and neglect, followed by emotional abuse and neglect. As previously discussed, these forms of abuse are the least closely related to suicide. However, it would still be advisable to assess traumatic history in migrant patients.

### Impulsivity

In this case, the data aligns with the controversial existing literature. On one hand, the placement of BIS-11 variables on the graph is more peripheral compared with trauma-related variables, PHQ-9 or SDQ, indicating their lesser influence. Specifically, only the BIS-11 motor subscale shows close direct relationships with different SDQ variables. No relationship was found with variables related to suicide attempts or variables associated with depression. However, the BIS-11 attentional subscale shows an inverse relationship between the SDQ and emotional neglect.

Based on the findings presented here, we deduce that the influence of impulsivity on suicide is not direct, however, it could potentially have an indirect influence through other variables such as the SDQ. It is important to note that BIS motor impulsiveness appears to exert the most influence, which aligns with the reviewed literature [14]. This may be due to several reasons. Motor impulsiveness involves acting on impulse without deliberation, which can lead to spontaneous and potentially dangerous behaviors, including self-harm and suicide attempts. Also when individuals experience intense emotional distress, those with high motor impulsivity may be more likely to engage in suicidal actions as a means of coping or escape.

### Drug consumption

Based on the presented graphs, the pathology related to alcohol and drug use does not show any relationship to variables associated with suicide attempts. Similarly, consumption of alcohol or drugs prior to the attempt does not appear to be related to the study variables.

As expected, substance use is related to itself, and alcohol-related pathology is associated with affective disorders (MINI-KID). Regarding centrality measures, all variables are situated at the bottom of the graph.

Based on our results, we conclude that the influence of substances in this case is minimal. Although some studies report the contrary [16, 18, 19], our results support the findings of some of the reviewed articles. For example, a large-scale American study conducted with a cohort of over 18,000 patients found that having an alcohol use disorder is a risk factor for young adults but not for adolescents. Additionally, they did not find any association between pathology related to the use of other substances and suicide, regardless of age [20]. One possible reason is that adolescents tend to consume alcohol and other substances in a more sporadic and experimental way. As a result, the secondary problems related to substance use may be more severe and frequent with long-term chronic use. Additionally, it is possible that the detection and diagnosis of substance use are underestimated, as the information is collected through interviews, which might reduce the honesty of responses, even without parents present. Finally, when interpreting these results, it is important to consider that the proportion of patients with a clinical diagnosis is small. As previously mentioned, the patients may still be too young to have developed full-blown substance-related pathology. In the future, it could be more valuable to collect information about consumption patterns, rather than focusing solely on established pathology.

### Influence on severity of suicidal behaviour

In relation to variables associated with severity of suicidal behaviour, the variable that shows the strongest relationships with others and has a more central position is the number of attempts variable. On the other hand, lethality of attempt is the least influential variable, as it does not show associations with any other variables beyond a correlation above 0.2. Another noteworthy finding is that the variables studied do not exhibit significant relationships with each other.

This association may have particular relevance in clinical practice for assessing the risk of suicide in patients who have already made a previous attempt. According to the results we obtained, higher scores on the SDQ scale (especially total scores) and higher scores on the PHQ-9 scale may be associated with a higher risk of reattempting, as they are correlated with a greater number of previous attempts. It is important to consider this when choosing a diagnostic support scale.

### Strengths and limitations

The results of this study must be interpreted in the context of certain limitations. First, the cross-sectional design of the study makes it difficult to to establish the direction of the findings. On the other hand, although the sample includes many patients from a clinical perspective, from a statistical point of view, it would be desirable to obtain a larger sample to achieve greater power. Another limitation is that the distribution by sex is very unbalanced, with very few patients identified as male. This, combined with the fact that it is a clinical population, could limit the extrapolation of the results to the general adolescent population. Another limitation to consider is the use of a version of the MINI-KID whose diagnostic criteria are based on the DSM-IV-TR. This makes the values outdated and complicates comparison with future research. Finally, another factor that complicates this comparison is the lack of studies conducted with the same methodology.

Despite the limitations described above, the study has several strengths to consider. One of the greatest strengths of the study is having a sample of patients in an understudied population, such as adolescent patients, using an innovative approach to the well-studied risk factors for suicide. Although the population under study is very specific, a relatively large sample has been achieved. This has been possible due to the collaboration among multiple centers across Spain. In addition to increasing the total sample size, this collaboration enhances its representativeness.

Another strength of the study is the methodology used to assess the adolescents. The evaluators were professionals with extensive experience in the field, and validated assessment instruments have been used, combining self-administered questionnaires with those administered by the evaluator. In addition, the assessment space provided confidentiality to create a trusting environment for the patient.

## Conclusion

Based on this study, we conclude that there are various risk factors associated with suicide that emerge during adolescence. Emotional and behavioural symptoms, as well as depression, exert the most influence on severity of suicidal behaviour and other factors. Specifically, they are closely related to total number of suicide attempts. Additionally, childhood trauma is associated with emotional difficulties, depression, and suicide. In terms of impulsivity, there is no relationship with variables associated with suicide, and only motor impulsiveness shows a direct relationship with the SDQ. The impact of other variables such as age, sex, and substance abuse seem to be less important than preiously published.

This article is proposed as a starting point for future research. It would be interesting to include additional risk factors, which would make the model more complex but likely better aligned with reality. It would also be crucial to conduct evaluations at different points in time to track changes and make comparisons, given that many of the factors are not static and may vary over time. The significant role of migration is noteworthy, opening the door for future research focused on social or demographic factors. Another intriguing finding is the limited impact of various diagnoses and the greater importance of self-reported values. This is particularly interesting as self-report measures are cost-effective and potentially more accessible, given that they are self-administered.

Taking all the aforementioned points into consideration, the use of questionnaires such as the SDQ or PHQ could be clinically useful when assessing suicide risk in adolescents.

## Electronic supplementary material

Supplementary Material 1

Supplementary Material 2

## Data Availability

No datasets were generated or analysed during the current study.
