# Supplementary figures and images for: Network analysis of influential risk factors in adolescent suicide attempters
Source: Child Adolesc Psychiatry Ment Health. 2024 Nov 25;18:152. doi: 10.1186/s13034-024-00842-9 (PMC11590239; doi:10.1186/s13034-024-00842-9)

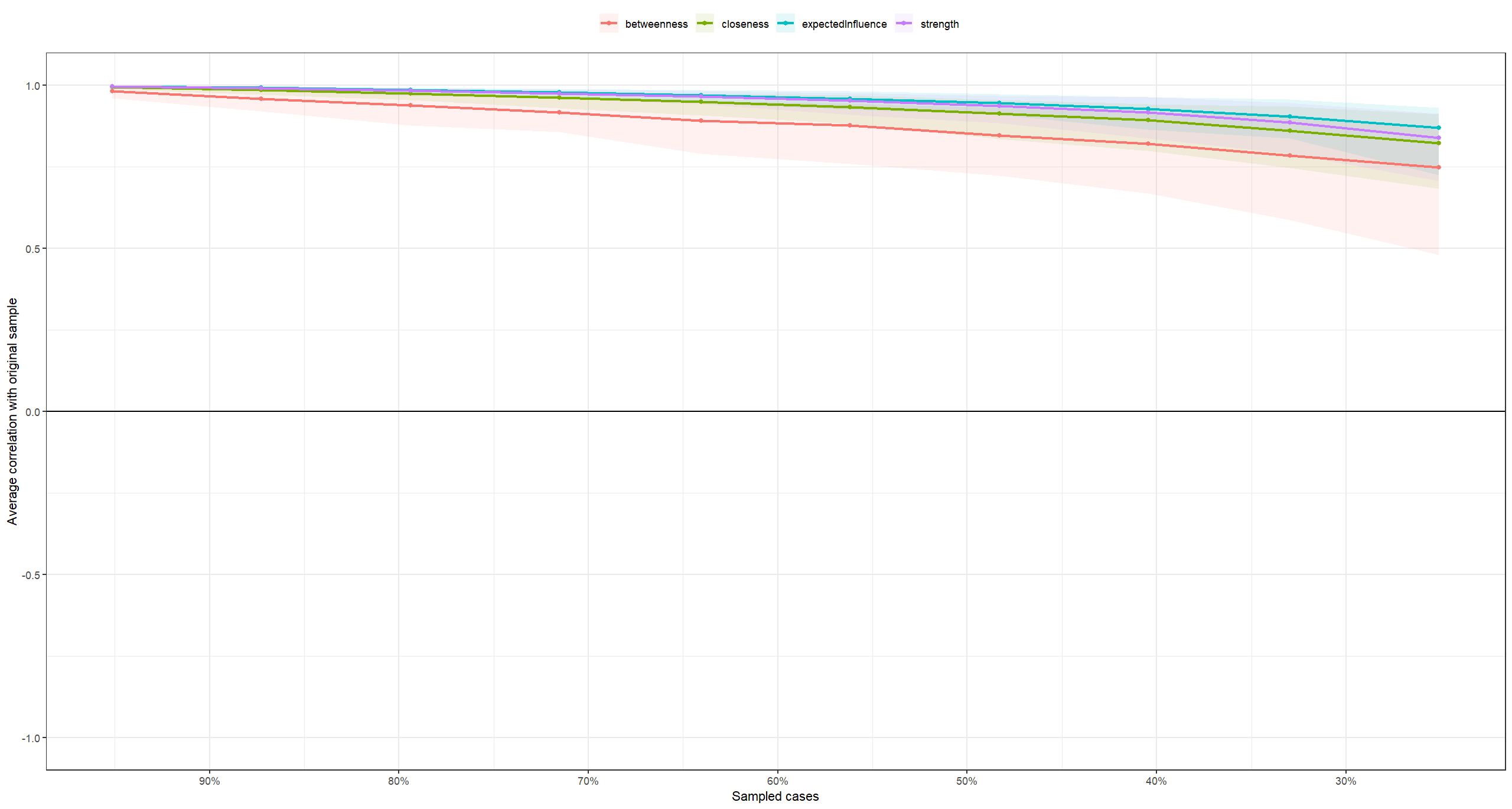

Supplement: Supplementary file 1 — Supplementary Material 1 [file 13034_2024_842_MOESM1_ESM.png]

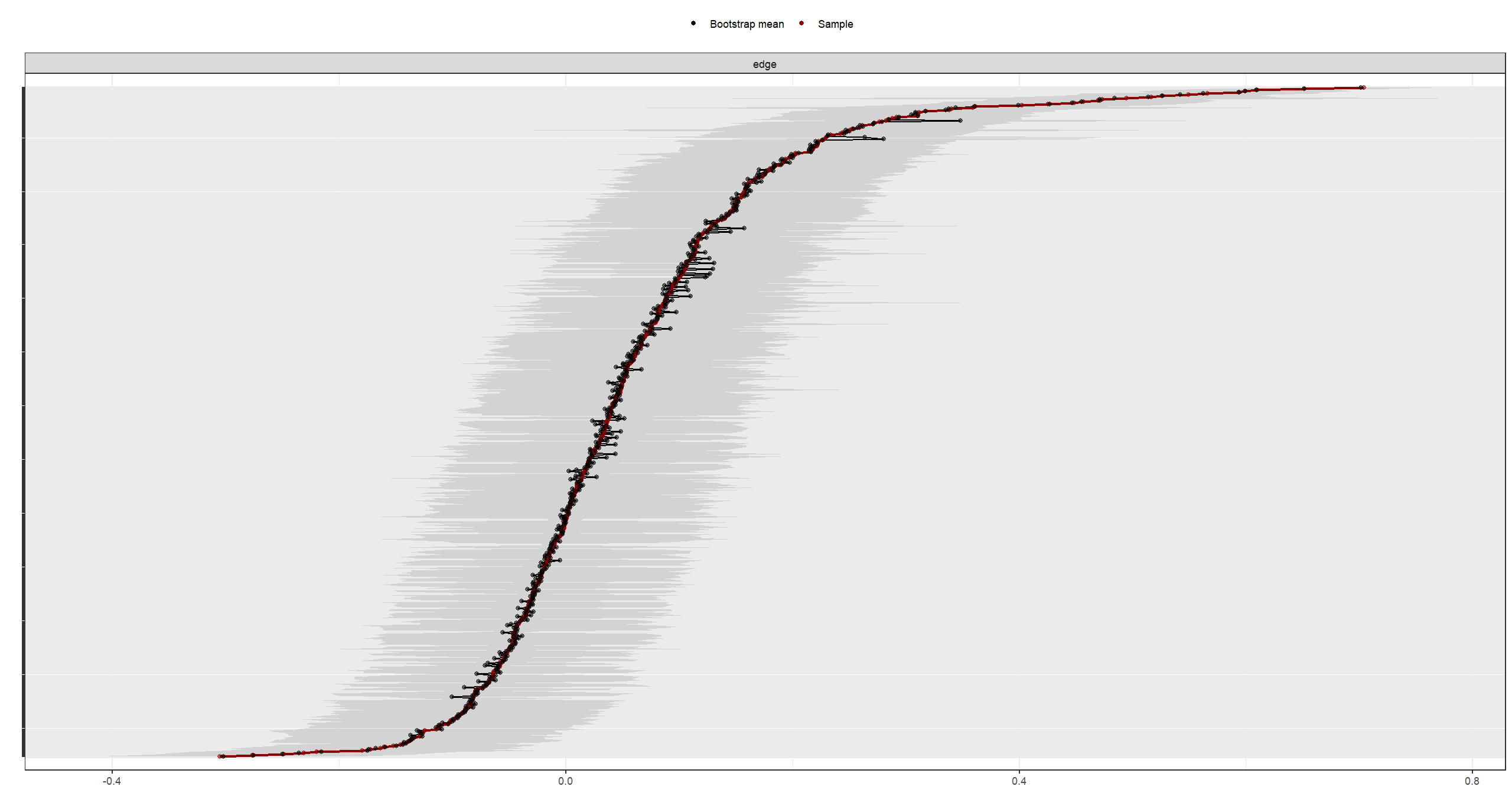

Supplement: Supplementary file 2 — Supplementary Material 2 [file 13034_2024_842_MOESM2_ESM.png]
